# Switchable Fluorescence of a Mechanical Stimulus-Responsive Au-P-S Complex

**DOI:** 10.3390/molecules29235736

**Published:** 2024-12-05

**Authors:** Jieqiong Huang, Yuanyuan Hu, Wendi Xu, Wei Yang, Chengrong Lu, David James Young, Zhi-Gang Ren

**Affiliations:** 1 Faculty of Environmental Engineering, Suzhou Polytechnic Institute of Agriculture, Suzhou 215008, China; 2 Suzhou Key Laboratory of Novel Semiconductor-Optoelectronics Materials and Devices, College of Chemistry, Chemical Engineering and Materials Science, Soochow University, Suzhou 215123, China; 3 Faculty of Food Science and Technology, Suzhou Polytechnic Institute of Agriculture, Suzhou 215008, China; 4 James Watt School of Engineering, University of Glasgow, University Avenue, Glasgow G12 8QQ, UK

**Keywords:** Ag-P-S complex, stimuli-responsive material, switchable luminescence, photoluminescent material

## Abstract

The reaction of [(3-bdppmapy)(AuCl)_2_] with NaHmna (3-bdppmapy = N,N’-bis-(diphenylphosphanylmethyl-3-aminopytidine, H_2_mna = 2-mercaptonicotinic acid)) resulted in a tetranuclear Au-P-S complex [(3-bdppmapy)_2_(AuHmna)_2_(AuCl)_2_] (**1**) which emitted bright yellow fluorescence at 542 nm under 377 nm excitation (QY = 5.3%, *τ* = 0.83 ns). Upon grinding, the emission intensity of **1** significantly and rapidly decreased, but could be recovered by exposure to CH_2_Cl_2_ vapor. This switchable fluorescence is attributed to the breaking and reforming of intermolecular hydrogen bonds with concomitant collapse and the restoration of the crystalline phase, which is not caused by static pressure or increased temperature.

## 1. Introduction

Coordination compounds constructed of Au(I) and phosphine and/or sulfur ligands can exhibit some interesting photophysical behaviors [1,2,3,4,5,6,7,8]. The strength of aurophilic Au···Au interactions and the rigidity of the molecules can vary in response to external stimuli, such as temperature, solvation, counter-ions and mechanical force, resulting in chromic and switchable photoluminescence (PL). This phenomenon has been applied to chemical sensing and information storage [8,9,10,11,12,13,14,15]. For example, the decanuclear Au(I) cluster [Au_10_{*μ*-Ph_2_PN(CH_2_Py)PPh_2_}_4_(*μ*_3_-S)_4_](PF_6_)_2_] crystallized in three different forms, each exhibiting blue, green, and red emissions, respectively, and displaying reversible PL to multiple stimuli including mechanical, steam, and heat [13].

Mechanical stimulus-responsive photoluminescent materials exhibit different emission colors or intensity in response to mechanical force, and this change may be reversed by exposure to certain solvents, steam, or heat [16,17,18,19,20,21,22,23]. This behavior has been attributed to changes in molecular stacking [24,25,26,27], changes in the length of Au···Au bonds [28,29,30,31], or changes in intermolecular hydrogen bonding, C-H···π, or π···π interactions [32,33,34]. The loss of volatile solvent molecules from gold complexes is also associated with changes in PL emissions [35,36,37]. Ito and co-workers reported the reversible luminescence of gold–isocyanide complexes associated with single-crystal-to-single-crystal phase transitions stimulated by mechanical cutting and solvent–vapor adsorption [36]. Mechanic forces usually destroy the crystalline phase and so it is hard to confidently identify the mechanism of mechanical stimulus response behavior in most cases.

We are interested in the design and applications of stimuli-responsive coinage metal complexes [38,39,40,41]. A Au(I) complex of diphosphine ligand 3-bdppmapy (N,N’-bis-(diphenylphosphanylmethyl)-3-aminopyridine) and sulfur ligand H_2_mba (2-mercaptobenzoic acid), [(3-bdppmapy)_2_(AuHmba)_3_(AuCl)] exhibited temperature-dependent emission [39]. We hypothesized that a different stimulus response could be achieved by replacing the sulfur ligand, and with this idea in mind, we mixed [(3-bdppmapy)(AuCl)_2_] with NaHmna (H_2_mna = 2-mercaptonicotinic acid) and obtained tetranuclear [(3-bdppmapy)_2_(AuHmna)_2_(AuCl)_2_] (**1**). Complex **1** exhibited PL quenching in response to mechanical grinding with the restoration of PL upon exposure to CH_2_Cl_2_ vapor. The phase transitions accompanying this PL switching were investigated. Herein, we report the preparation, structure, and mechanism of the switchable PL of **1**.

## 2. Results and Discussion

### 2.1. Synthesis and Characterization

**Scheme 1:**
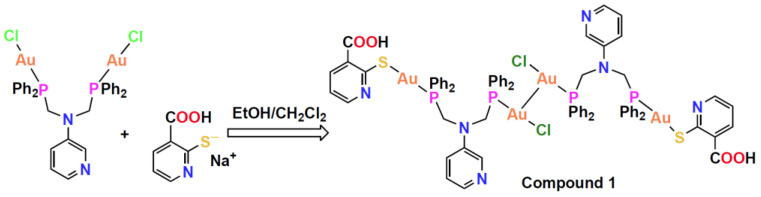
Synthesis of compound **1**.

The reaction of [(3-bdppmapy)(AuCl)_2_] with NaHmna (molar ratio 1:1) in CH_2_Cl_2_/EtOH at room temperature followed by the diffusion of the product solution with Et_2_O produced single crystals of **1**·0.5Et_2_O·0.5CH_2_Cl_2_ in 68% yield (Scheme 1). Reactions using other ratios of reactants did not improve the yield. The TGA of **1**·0.5Et_2_O·0.5CH_2_Cl_2_ (Figure S1) revealed the loss of lattice solvent molecules above room temperature. The main structure remained stable below 200 °C, which subsequently lost the organic species (−Ph, −Py and Hnma^−^) at higher temperatures (200–360 °C, weight loss of 50%, calcd. of 51%).

Compound **1** was stable towards air and water, soluble in DMF and DMSO, and insoluble in other common solvents. The IR spectrum of **1** (Figure S2) contained an absorption at 3053 cm^−1^ attributed to the stretching vibration of −OH in Hmna^−^, indicating that the −COOH group was not deprotonated. The ^1^H NMR spectrum in DMSO-*d*_6_ (Figure S3) contained signals for the −CH_2_− (5.09 ppm, 8H), −Ph, −Py (6.85, 6.93, 7.29–8.22 ppm, 54H), and −COOH (12.94 ppm, 2H) protons. The ^31^P{^1^H} NMR spectrum contained a single resonance for the −PPh_2_ group at 21.08 ppm.

**Figure 1:**
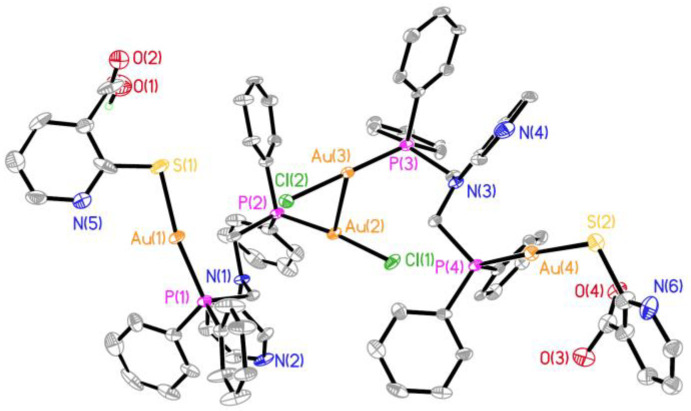
Crystal structure of **1**·0.5Et_2_O·0.5CH_2_Cl_2_ with 30% thermal ellipsoids. Solvent molecules and H atoms (except for those of the −COOH group) are omitted for clarity.

A single crystal X-ray diffraction (SCXRD) analysis indicated that complex **1**·0.5Et_2_O·0.5CH_2_Cl_2_ crystallized in the triclinic space group *P*ī, and its asymmetric unit consisted of one [(3-bdppmapy)_2_(AuHmna)_2_(AuCl)_2_] molecule, a half disordered Et_2_O, and a half disordered CH_2_Cl_2_ molecule. The Au(I) atoms were almost linearly coordinated by a −PPh_2_ group of 3-bdppampy and the S atom of the Hmna^−^ anion (Au1 and Au4) or a Cl^−^ anion (Figure 1 and Table S2). The P−Au−S and P−Au−Cl angles were 169.89(11) and 177.37(8)°, respectively. The Au2−Au3 distance (3.1312(6) Å) was consistent with an aurophilic interaction. The mean Au−P, Au−S, and Au−Cl bond lengths (2.241(2), 2.302(3) and 2.293(2) Å, respectively) were in the normal ranges. All the N atoms in 3-bdppmapy and in Hmna^-^ remained uncoordinated, while the two −PPh_2_ groups of each 3-bdppmapy bridged two Au atoms.

### 2.2. Photophysical Properties

**Figure 2:**
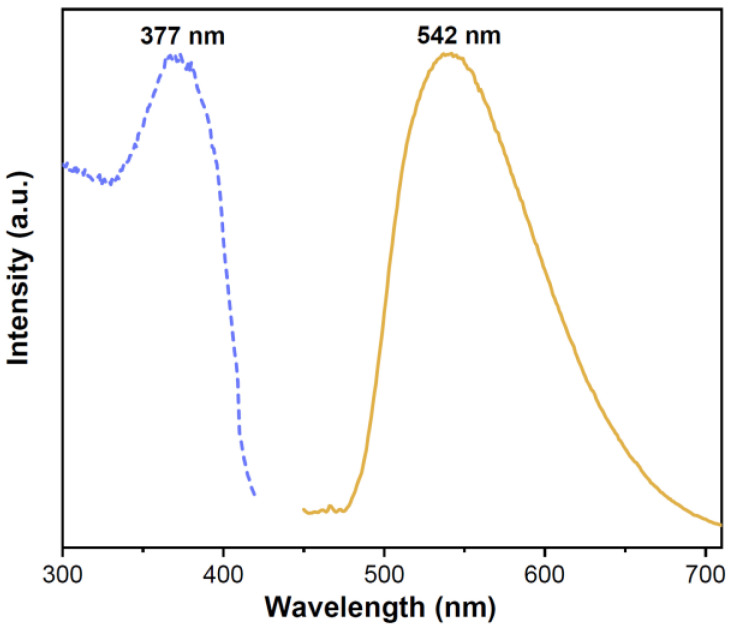
Solid-state (left) excitation and (right) emission spectra of compound **1** at room temperature.

The solid-state photoluminescence of **1** was measured at an ambient temperature. Upon excitation of 377 nm, **1** emitted yellow at 542 nm (Figure 2). The quantum yield (QY) was 5.3% with a lifetime (*τ*, excited at 370 nm, Figure S4) of 0.83 ns, which indicated that the emission was due to fluorescence. The QY was comparable with other reported Au-P complexes (listed in Table S1), while the nanosecond scale lifetime could be found in the Au complexes with 3-bdppmapy [38,39], and were somewhat rare in other reported Au-P complexes. Density functional theory (DFT) calculations based on the SCXRD data were used to calculate the frontier orbital distributions. The HOMOs were mainly located on the π orbitals of one Hnma^−^ anion and the −Py groups in 3-bdppmapy (Figure S5), while the LUMOs were located at the π* orbitals of the other Hnma^−^ anion and the −PPh_2_ groups in 3-bdppmapy. The excitation and emission were therefore likely due to a ligand-to-ligand charge transfer (LLCT).

**Figure 3:**
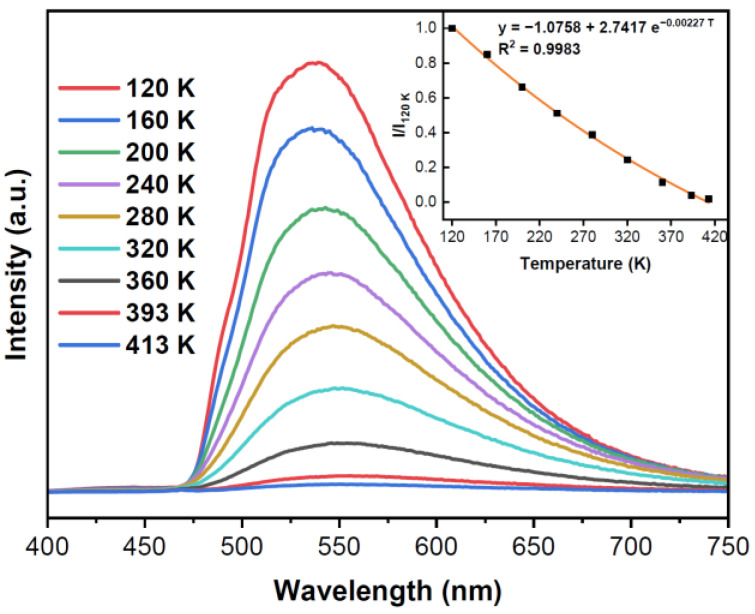
Emission spectra of **1** at 120 to 413 K (Ex = 377 nm). Inset was the ratios of the maximum intensities compared to that at 120 K.

Measuring the temperature-dependent emission of **1** from 120 K up to 413 K (Figure 3) revealed that the maximum emission intensity gradually decreased with the increasing temperature in an approximately linear relationship over specific temperature windows, *I*/*I*_120 K_ = −1.0758 + 2.7417 e^−0.00227 T^ (T = 120 to 413 K, *R*^2^ = 0.9983), accompanied by a minor shift in the λ_max_ (537 nm at 120 K). The lifetime *τ* at 80 K (excited at 370 nm, Figure S6) was 1.6 ns. This enhancement of the emission intensity and longer lifetime at lower temperatures was attributed to the restriction of the intramolecular rotation (RIR) of the −Ph and −Py groups that suppressed non-radiative decay. Consistent with this finding was the observation that both the DMF and DMSO solutions of **1** were almost non-emissive (Figure S7), presumably due to enhanced rotation of the −Ph and −Py groups in these aprotic solvents.

### 2.3. Switchable Fluorescence of 1 in Response to Mechanical Stimulus

**Figure 4:**
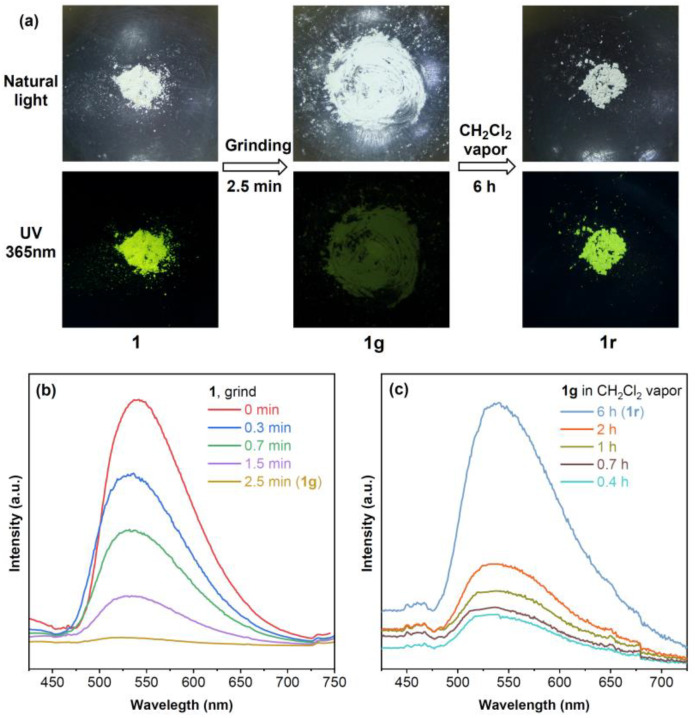
(**a**) Photographs of samples of **1**, **1g** and **1r** under natural light (upper) and under 365 nm UV light (lower). The emission spectra of (**b**) **1** after grinding for different times, and (**c**) **1g** after exposure to CH_2_Cl_2_ vapor for different times (Ex = 377 nm).

Grinding the crystals of **1** in a mortar decreased the emission intensity of this complex (Figure 4a). The emission after 2.5 min of grinding (**1g**) shifted slightly to 520 nm (Figure 4b) and the QY reduced to 0.35%, which was 1/15th of that of **1**.

**Figure 5:**
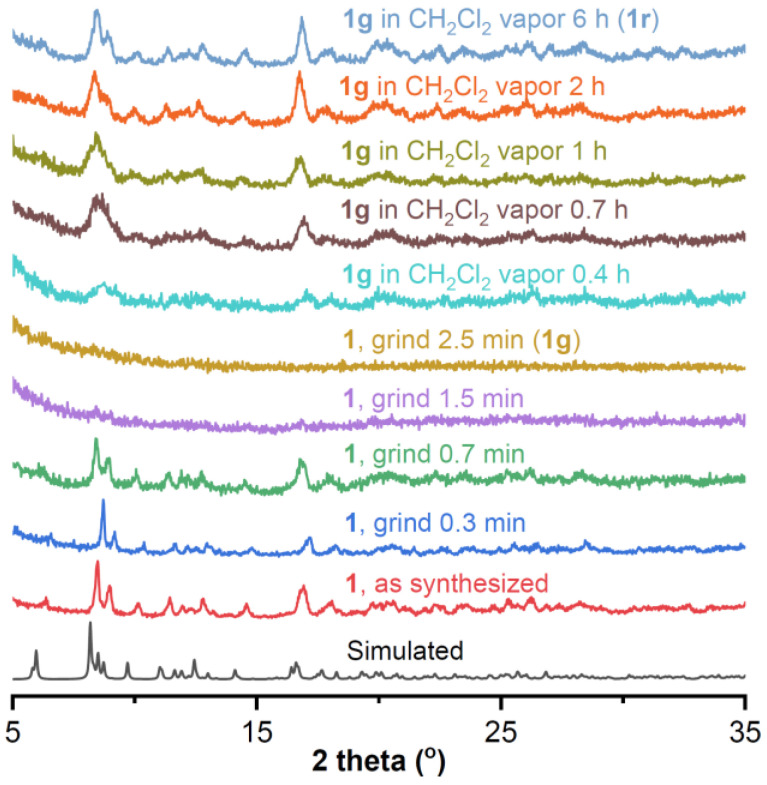
PXRD patterns of **1** simulated from the SCXRD data, as synthesized, after grinding for different times, and of **1g** after exposure to CH_2_Cl_2_ vapor for different times.

The PXRD patterns of **1** (Figure 5) and those heating below the melting temperature (150 °C, Figure S8) indicated similar crystalline phases comparable to that simulated from the SCXRD data, demonstrating that the packing mode was not affected by the elimination of lattice solvent. The major peaks in the PXRD pattern of **1** shifted slightly in the first 0.7 min of grinding, and became unrecognizable after 1.5 min, indicating that **1g** was amorphous. In addition, when a powdered sample of **1g** was exposed to CH_2_Cl_2_ vapor for 6 h, the emission of the resulting solid (**1r**) gradually recovered (Figure 4b). The PXRD pattern of this material also recovered and was similar to that of **1**. In comparison, the fluorescence of **1g** was not recoverable after placing in Et_2_O vapor for 2 h (Figure S9), indicating the recovery was selectively toward solvent.

In the IR spectra (Figure S10), the vibrations of the −Ph and −Py groups at 1263 and 1691 cm^−1^ of **1** were shifted slightly to 1242 and 1711 cm^−1^ in **1g**, and then reverted back to their original values in **1r**. These PXRD and IR spectroscopic experiments indicated that **1** and **1r** were of a similar crystalline phase. As shown in Figure S11, the emission of **1** was only slightly weakened over five interconversion cycles between **1** and **1g** using mechanical force and exposure to CH_2_Cl_2_ vapor.

The solid-state UV-Vis spectra of **1** and **1g** (Figure S12) showed similar absorption bands. Likewise, most of the strong IR peaks of **1** and **1g** were similar, indicating the molecular structure of **1** remained unchanged during grinding. When the solvent molecules were removed from the crystal structure of **1**·0.5Et_2_O·0.5CH_2_Cl_2_, there was a total void calculated to be 303 Å^3^ in each cell (8.1% of the cell volume, calculated using PLATON [42], Figure S13). Hence, we believe that the basic packing mode of **1**·0.5Et_2_O·0.5CH_2_Cl_2_ remained unchanged in **1** when the solvent molecules were removed, but collapsed under grinding and caused the reversible transformation between **1** (**1r**) and **1g**.

**Figure 6:**
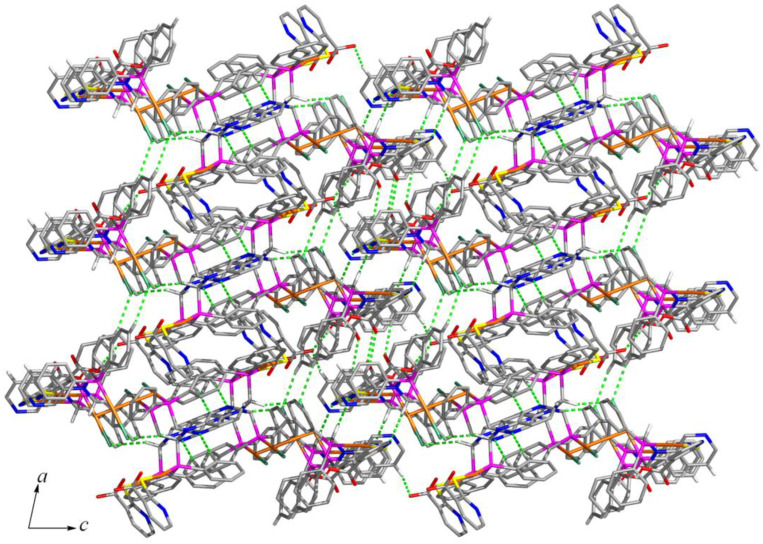
Packing of the main structure of **1** viewed along the *b* axis in the crystal structure of **1**·0.5Et_2_O·0.5CH_2_Cl_2_. Hydrogen bonds were plotted as green dash lines. Atom colors: Au, orange; S, yellow; P, purple; Cl, green; N, blue; O, red; C, dark gray; H, light gray.

Further analysis of the SCXRD data indicated seven intermolecular and one intramolecular hydrogen bond in the crystal structure of **1**·0.5Et_2_O·0.5CH_2_Cl_2_ (Table 1, Figure 6 and Figure S14), of which two C−H···O, two C−H···N, and one C−H···Cl bonds involved the −Ph and −Py groups. These abundant intermolecular hydrogen bonds maintained the packing when the solvent molecules were removed from **1**·0.5Et_2_O·0.5CH_2_Cl_2_, but were disrupted by grinding resulting in the collapse of the crystalline phase. Consequently, the rotation of the −Ph and −Py groups that were restricted in **1** could rotate more freely in **1g** when these hydrogen bonds were disrupted and therefore switched off fluorescence in **1g**.

Pressure, temperature, and molecular sliding were the major aspects among the mechanical grinding. When **1** was subjected to static pressure (12 MPa, 9 min), the emission wavelength of the resulting solid (**1p**) remained unchanged (Figure S15). Likewise, the PXRD pattern (Figure S8) and IR spectrum (Figure S10) were very similar to those of **1**, indicating that the crystalline phase of **1** remained unchanged and did not convert to **1g** in this case.

**Figure 7:**
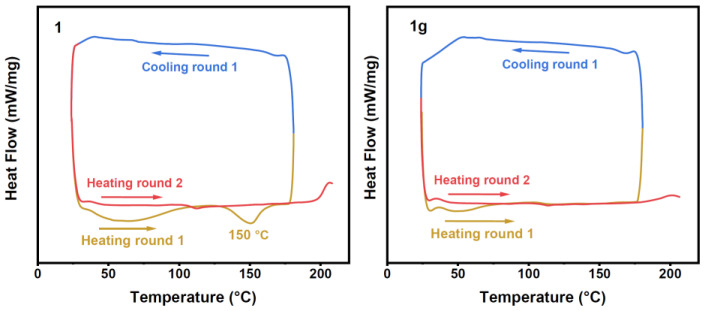
DSC heat flow curves of (**left**) **1** and (**right**) **1g** in the first two heating rounds. The first round was heating to 180 °C and then cooling to room temperature, while the second round involved reheating to 205 °C.

The DSC curves of **1** and **1g** were then measured to better understand the influence of temperature. As shown in Figure 7, during the first heating round (room temperature to 180 °C), compound **1** lost the residual solvent molecules below 100 °C, and melted in the range 135–165 °C (centered at 150 °C). Cooling to room temperature provided a solid which showed no obvious decalescence peak at around 150 °C in the second heating round, indicating this new phase was different to **1**. By comparison, **1g** showed no decalescence peak at 150 °C in both the first and second heating rounds. The curves of **1** and **1g** in the second heating round (room temperature to 230 °C) were quite similar, and their decomposition exothermic peaks were both around 205 °C, indicating that **1g** likely possessed a similarly collapsed solid phase to that of melted and re-solidified **1** (designated **1m**). However, the possible role of local-area melting during grinding was ruled out because grinding **1** at a lower temperature (−40 °C) still led to the formation of **1g**.

Was the heating below the melting temperature able to prompt the collapse of the crystalline phase? The emission spectra of **1** measured at 120 °C (393 K, designated **1**–**120**) and at 140 °C (413 K, designated **1**–**140**) was compared with **1m**. A sample (**1mw**) of treating **1** with microwave (2450 MHz, 700 W, 5 min) was also loaded in comparison because the microwave is helpful in exacerbating the thermal motion of molecules at room temperature. All of the treated samples had similar emission spectra to that of **1** (Figure S16). Sample **1m** emitted significantly reduced fluorescence, comparable in intensity to that of **1g**. By comparison, **1mw** exhibited emissions of similar wavelength and intensity to that of **1**. The decreased intensity of **1**–**120** and **1**–**140** is attributable to enhanced thermal motions facilitating non-radiative decay. The PXRD pattern (Figure S8) of **1m** indicated it was amorphous, while those of **1p**, **1**–**120**, **1**–**140** and **1mw** were similar to that of **1**. The IR spectrum of **1m** (Figure S10) revealed that the absorptions at 1242 and 1711 cm^−1^ were identical to the corresponding signals in the IR spectrum of **1g**, but were shifted to 1263 and 1691 cm^−1^ in **1p**, **1–120**, **1–140,** and **1mw**. These results indicate that **1** remained crystalline at higher temperatures (**1**–**120** and **1**–**140**) and after microwave treatment (**1mw**), but collapsed to an amorphous material after grinding (**1g**) or melting (**1m**).

These observations lead us to speculate that the molecules in crystalline **1** slid and filled the lattice voids created during grinding and melting. Concurrently, the intermolecular hydrogen bonds were disrupted, resulting in greater freedom for the aromatic groups pendant on the ligand, which increased the non-radiative transitions and the quenching of the fluorescence of **1**. In addition, the recovering phase transition from **1p** to **1r** (**1**) was due to the CH_2_Cl_2_ molecule reoccupying the voids in **1p**, as some of us have previously reported that the crystalline structure of the Au-P complex was restored upon exposure to CH_2_Cl_2_ vapor [38].

## 3. Experimental Section

### 3.1. Materials, Characterization, and Measurements

3-Bdppmapy was prepared by a literature method [43]. All the other reagents were used directly from commercial sources without further purification. The elemental analysis (EA) of C, H and N was performed on a Carlo-Erba CHNO-S microanalyzer (Carlo Erba Instruments, Milan, Italy). PXRD patterns were obtained on a Bruker D2 PHASER diffractometer (Bruker Instruments, Karlsruhe, Germany) using a Cu-*K*_α_ (*λ* = 1.54056 Å) incident light source at 30 kV and 10 mA. Infrared spectra were measured on a Bruker Tensor 27 FT-IR spectrometer with ATR equipment (4000−500 cm^−1^, Bruker Instruments, Karlsruhe, Germany). NMR spectra were acquired on a Varian UNITY plus-400 NMR spectrometer (Varian Medical Systems, Palo Alto, CA, USA) at room temperature. Chemical shifts (*δ*, ppm) were referenced to the solvent signal of DMSO-*d*_6_ for ^1^H and ^13^C NMR and 85% H_3_PO_4_ for ^31^P NMR, respectively. The thermogravimetric analysis (TGA) data were measured on a TA SDT-Q600 analyzer (TA Instruments, New Castle, DE, USA) at a heating rate of 10 °C·min^−1^ in N_2_ flow. The emission spectra, transient photoluminescence, and quantum yields were measured on a FLS1000 (Edinburgh Instruments, Livingston, UK) spectrometer. The UV/Vis spectra were recorded on a Shimadzu UV-3600 spectrophotometer (Shimadzu Corporation, Kyoto, Japan).

### 3.2. Synthesis of [(3-bdppmapy)_2_(AuHmna)_2_(AuCl)_2_] (**1**)

[(3-bdppmapy)(AuCl)_2_] was prepared in situ by stirring 3-bdppmapy (0.0062 g, 0.0125 mmol) and Au(tht)Cl (0.0080 g, 0.025 mmol) for 3 h in CH_2_Cl_2_ [38]. H_2_mna (0.0019 g, 0.0125 mmol) dissolved in 0.25 mL EtOH was mixed with 0.05 M NaOH/EtOH (0.25 mL, 0.0125 mmol) to obtain a light yellow solution of NaHmna. The solutions of the metal and ligand were then mixed, stirred at room temperature for 3 h, and filtered to afford a colorless filtrate. Colorless crystals of **1**·0.5Et_2_O·0.5CH_2_Cl_2_ were obtained by slow diffusion with Et_2_O for one week. These crystals were dried in vacuo to remove solvent molecules. The yield of **1** was as follows: 9.1 mg (68% based on Au). The anal. calcd for C_74_H_64_Au_4_Cl_2_N_6_O_4_P_4_S_2_ were C, 41.38; H, 3.00; N, 3.91. The following was found: C, 40.93; H, 3.26; N, 3.89 (%). The IR was as follows (ATR, cm^−1^): 3053 (w), 2939(w), 2162(w), 1979 (w), 1691 (w), 1609(w), 1566 (m), 1483 (m), 1435 (s), 1379 (s), 1263 (m), 1217 (m), 1184 (w), 1124 (m), 1103 (s), 1061 (m), 997 (w), 868 (s), 815 (w), 793 (w), 745 (vs), 721 (s), 689 (vs), 648 (w). The ^1^H NMR (400 MHz, DMSO-*d*_6_, 298 K, ppm) *δ* = 12.94 (brs, 2H), 8.22 (dd, *J* = 4.8, 1.9 Hz, 2H), 8.17 (d, *J* = 2.9 Hz, 2H), 7.72−7.86 (m, 18H), 7.50−7.57 (m, 10H), 7.41−7.49 (m, 16H), 7.29−7.35 (m, 2H), 6.93 (dd, *J* = 7.6, 4.8 Hz, 2H), 6.85 (dd, *J* = 8.4, 4.6 Hz, 2H), 5.09 (s, 8H). The ^13^C NMR (101 MHz, DMSO-*d*_6_, 298 K, ppm) was as follows: *δ* = 168.33, 167.46, 149.05, 142.93, 141.06, 140.69, 136.94, 133.94, 133.81, 132.06, 132.03, 129.18, 129.07, 129.02, 128.28, 127.75, 125.34, 122.82, 117.43, 54.91, 31.35, 50.99. The ^31^P NMR was as follows (162 MHz, DMSO-*d*_6_, 298 K, ppm): *δ* = 21.08 (s).

### 3.3. Single-Crystal X-Ray Diffraction (SCXRD) Determination

A single crystal of **1**·0.5Et_2_O·0.5CH_2_Cl_2_ was selected directly from the diffusion tube. A single crystal X-ray diffraction (SCXRD) analysis was performed on a Bruker D8 venture CCD diffractometer (Bruker Instruments, Karlsruhe, Germany) using Ga *K*_α_ (*λ* = 1.34138 Å) radiation at 110 K. The diffraction data were processed by Bruker SAINT and multi-scan absorption correction [44,45]. The structure was solved by direct methods using SHELXS and refined by full-matrix least-squares methods against *F*^2^ by SHELXL [46]. The Et_2_O and CH_2_Cl_2_ solvent molecules were disordered over two sites with occupancies 0.5/0.5, which were refined isotropically without adding H atoms. All the other non-hydrogen atoms were refined anisotropically, while all the other hydrogen atoms were obtained via theoretical hydrogenation. Selected crystallographic data and refinement parameters are listed in Table S2. Selected bond lengths and angels are listed in Table S3.

### 3.4. Computational Details

Theoretical calculations were conducted using the Gaussian 16 (Rev C.01) software package at the B3LYP-GD3BJ/def2SVP level.

## 4. Conclusions

We have prepared a new tetranuclear Au-P-S complex **1** via the reaction of [(3-bdppmapy)(AuCl)_2_] with NaHmna. Complex **1** emitted bright green fluorescence at 542 nm under 377 nm excitation with a QY of 5.3%. The fluorescence of **1** was significantly quenched (QY = 0.35%) by grinding for 2.5 min, and this fluorescence recovered slowly upon exposure to CH_2_Cl_2_ vapor. This on–off switching was repeatable and related to a phase transition caused by the collapse and restoration of the crystalline phase of **1** attributed to the disruption and reforming of intermolecular hydrogen bonds, which in turn caused the restriction of intramolecular rotation (RIR) of the ligand −Ph and −Py groups. Removing these restrictions on the free rotation of these groups increased the non-radiative decay of the excited molecules. We propose that this phase transition on grinding resulted in the sliding of molecules that did not occur when the crystals of **1** were subjected to static pressure or increased temperature. While the switching of the coinage metal complex photoluminescence using mechanical force has been reported often in the past, the underlying mechanism for this switching has not hitherto been elucidated. The results above are a step forward in this respect. Non-covalent interactions in the solid-state can be adjusted by altering the chemical environment with a concomitant change in photoluminescence, and this could be realized by selecting appropriate ligands in a general strategy for the design of new sensing materials.

## Figures and Tables

**Table 1:** Hydrogen bond lengths (Å) and angles (°) in **1**·0.5Et_2_O·0.5CH_2_Cl_2_.

D−H···A	D−H	H···A	D···A	D−H···A
O3−H3···O2 ^i^	0.84	1.74	2.579 (13)	131
C1−H1A···Cl2	0.99	2.76	3.634 (9)	147
C1−H1B···N2 ^ii^	0.99	2.59	3.569 (13)	169
C13−H13···N2 ^ii^	0.95	2.51	3.451 (16)	172
C33−H33B···N6 ^iii^	0.99	2.47	3.408 (14)	157
C35−H35···O4 ^iv^	0.95	2.26	3.139 (13)	154
C36−H36···O2 ^v^	0.95	1.70	2.633 (12)	166
C42−H42···Cl2 ^v^	0.95	2.77	3.601 (12)	146

Symmetry codes: (i) −1 + x, 1 + y, z; (ii) 1 − x, 2 − y, −z; (iii) 1 − x, 2 − y, 1 − z; (iv) −x, 2 − y, 1 − z; (v) −1 + x, y, z.

## Data Availability

The crystallographic data are available from the Cambridge Crystallographic Data Centre (CCDC number 2382709). Other data not presented in the Supplementary Materials are available on request from the corresponding author.

## Supplementary Materials

The following supporting information can be downloaded at https://www.mdpi.com/article/10.3390/molecules29235736/s1; Figure S1: TGA curves of **1**·0.5Et_2_O·0.5CH_2_Cl_2_; Figure S2: IR spectra of **1**; Figure S3: ^1^H, ^13^C and ^31^P NMR spectra of **1**; Figure S4: transient photoluminescence data of **1** at 298 K; Figure S5: orbital distributions of HOMOs and LUMOs in **1**; Figure S6: transient photoluminescence data of **1** at 80 K; Figure S7: emission spectra of **1** and the DMSO and DMF solutions of **1**; Figure S8: the PXRD patterns of as synthesized **1**, **1p**, **1** heated at 120 °C (**1–120**) and 140 °C (**1–140**), **1m** and **1mw**; Figure S9: photographs of **1**, **1g,** and **1g** in Et_2_O vapor for 2h under natural light and under 365 nm UV light, and their emission spectra; Figure S10: comparison of the IR spectra in the range of 1000–2000 cm^−1^ of **1**, **1g**, **1r**, **1p**, and **1**, heated at 120 °C (**1–120**) and 140 °C (**1–140**), **1m** and **1mw**; Figure S11: photo pictures of **1** and **1g** under irradiation at 365 nm over five interconversion cycles; Figure S12: solid-state UV-Vis spectra of **1** and **1g**; Figure S13: plot representing the voids in the single crystal of **1**·0.5Et_2_O·0.5CH_2_Cl_2_ after removing all the solvates; Figure S14: packing of the main structure of **1** viewed along the *a* axis in the crystal structure of **1**·0.5Et_2_O·0.5CH_2_Cl_2_; Figure S15: emission spectra of **1** and **1p**; Figure S16: emission spectra of **1m**, **1mw**, **1** at 280 K, at 393 K (**1–120**) and at 413 K (**1–140**); Table S1: emission wavelength (λ_max_), life time (τ) and quantum yield (QY) of some Au-P complexes in the literature; Table S2: selected crystallographic data and refinement parameters for **1**·0.5Et_2_O·0.5CH_2_Cl_2_; Table S3: selected bond lengths and angels in **1**·0.5Et_2_O·0.5CH_2_Cl_2_.
