# Supplementary material for: Switchable Fluorescence of a Mechanical Stimulus-Responsive Au-P-S Complex
Source: Molecules. 2024 Dec 5;29(23):5736. doi: 10.3390/molecules29235736 (PMC11643574; doi:10.3390/molecules29235736)
Supplement: Supplementary file 1 [file molecules-29-05736-s001.zip › molecules-3303894-supplementary.pdf]

Contents

Figure S1. TGA curve of **1**·$0.5Et_2O·0.5CH_2Cl_2$ ···· S1

Figure S2. IR spectrum of compound **1** ···· S1

Figure S3. ^1H, ^{13}C and ^{31}P NMR spectra of **1** in DMSO-d_6 ···· S2

Figure S4. Transient photoluminescence data of **1** at 298 K ···· S3

Figure S5. Orbital distributions of HOMOs and LUMOs in **1** ···· S3

Figure S6. Transient photoluminescence data of **1** at 80 K ···· S4

Figure S7. Emission spectra of **1** and the DMSO and DMF solutions of **1** ···· S4

Figure S8. The PXRD patterns of as synthesized **1**, **1p**, **1** heated at 120 °C (**1–120**) and 140 °C (**1–140**), **1m** and **1mw**. ···· S4

Figure S9. Photographs of **1**, **1g** and **1g** in Et_2O vapor for 2h under natural light and under 365 nm UV light, and their emission spectra. ···· S5

Figure S10. Comparison on the IR spectra in the range of 1000–2000 cm^{-1} of **1**, **1g**, **1r**, **1p**, **1** heated at 120 °C (**1–120**) and 140 °C (**1–140**), **1m** and **1mw** ···· S5

Figure S11. Photo pictures of **1** and **1g** under irradiation at 365 nm over five interconversion cycles ···· S6

Figure S12. Solid-state UV-Vis spectra of **1** and **1g** ···· S6

Figure S13. Plot representing the voids in the single crystal of **1**·$0.5Et_2O·0.5CH_2Cl_2$ after removing all solvates ···· S7

Figure S14. Packing of the main structure of **1** viewed along the *a* axis in the crystal structure of **1**·$0.5Et_2O·0.5CH_2Cl_2$ ···· S7

Figure S15. Emission spectra of **1** and **1p** ···· S8

Figure S16. Emission spectra of **1m**, **1mw**, **1** at 280 K, at 393 K (**1–120**) and at 413 K (**1–140**) ···· S8

Table S1. Emission wavelength (λ_{max}), life time (τ) and quantum yield (QY) of some Au–P complexes in literature ···· S9

Table S2. Selected crystallographic data and refinement parameters for **1**·$0.5Et_2O·0.5CH_2Cl_2$ ···· S9

Table S3. Selected bond lengths and angels in **1**·$0.5Et_2O·0.5CH_2Cl_2$ ···· S10

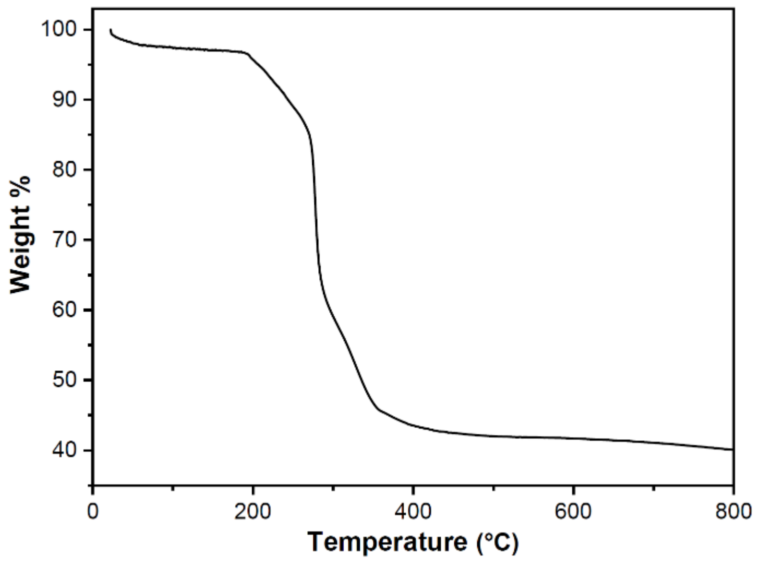

Figure S1. TGA curve of **1**·0.5Et_2O·0.5CH_2Cl_2 from room temperature to 800 °C in a N_2 stream.

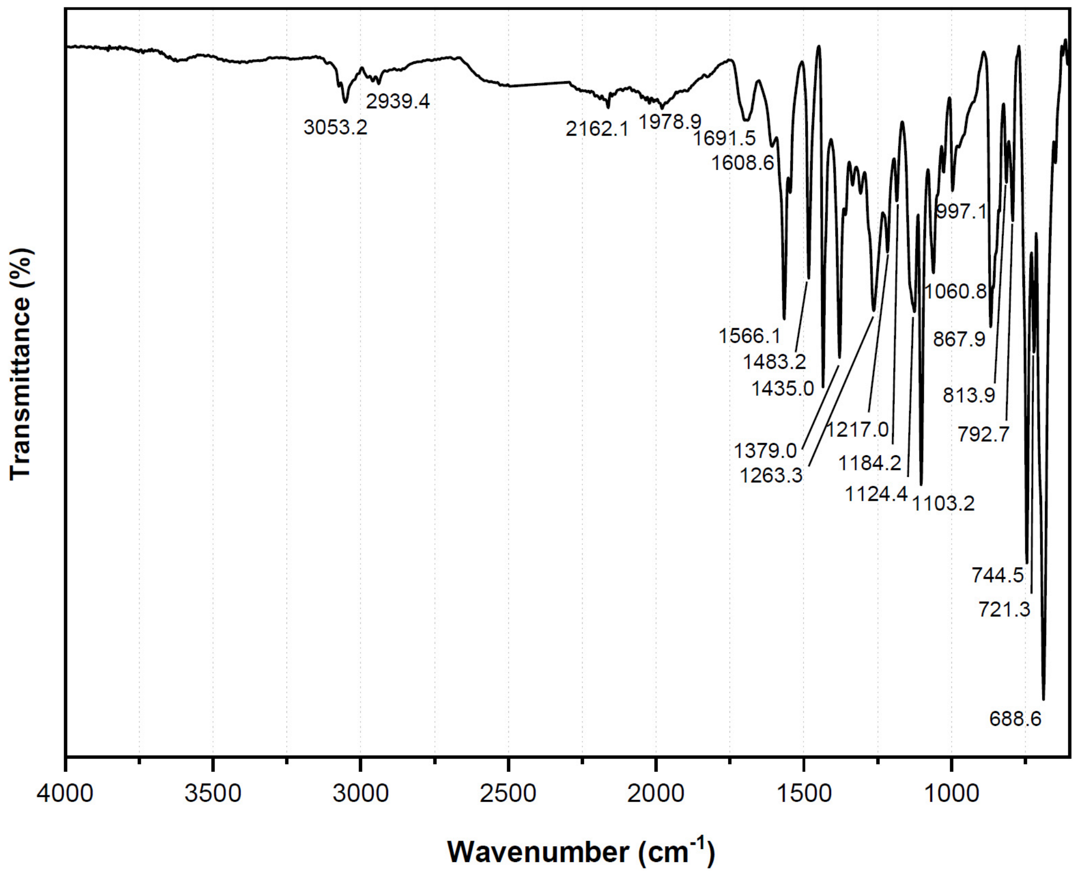

Figure S2. IR spectrum of compound **1.**

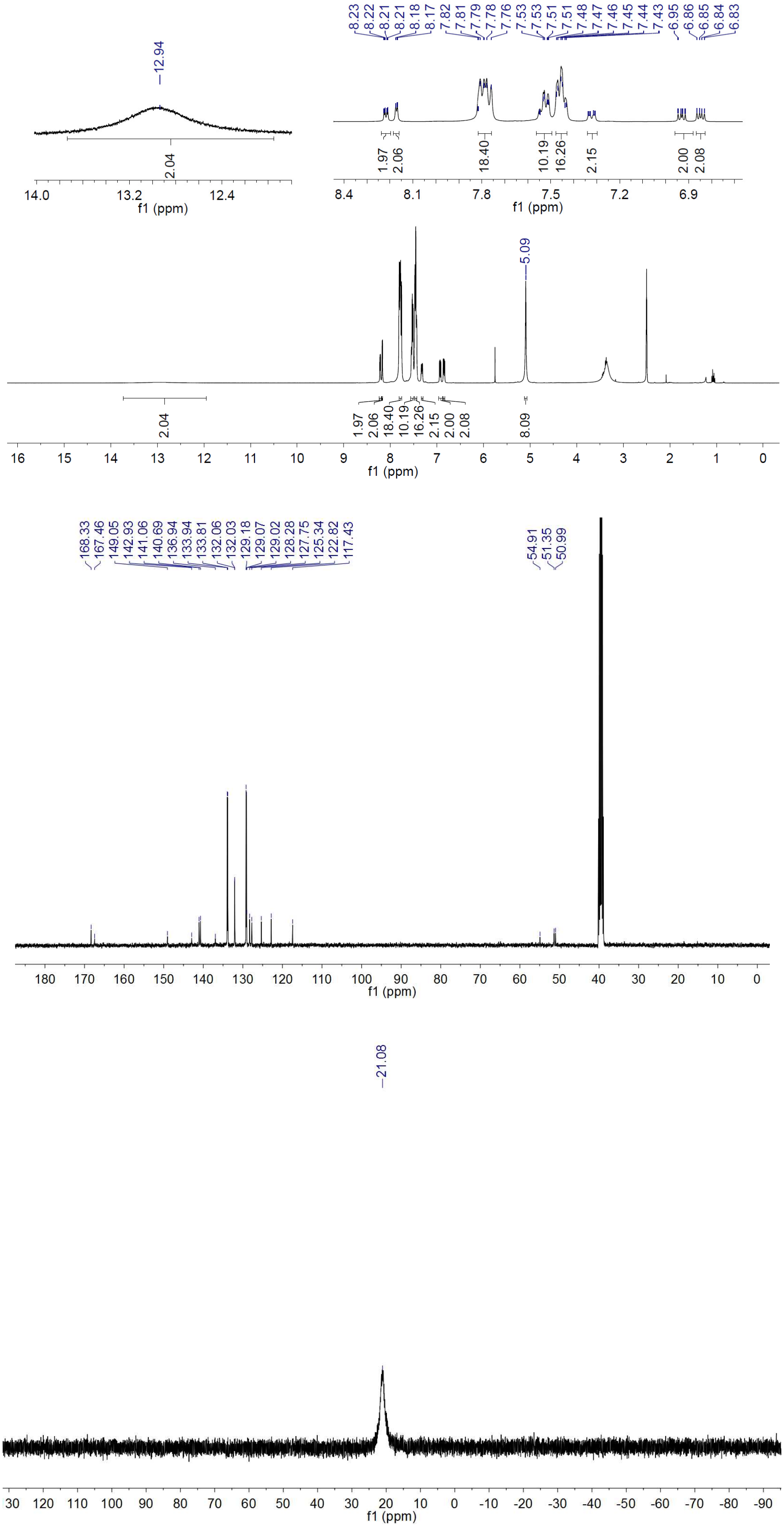

Figure S3. ^{1}H, ^{13}C and ^{31}P NMR spectra of **1** in DMSO-d_6.

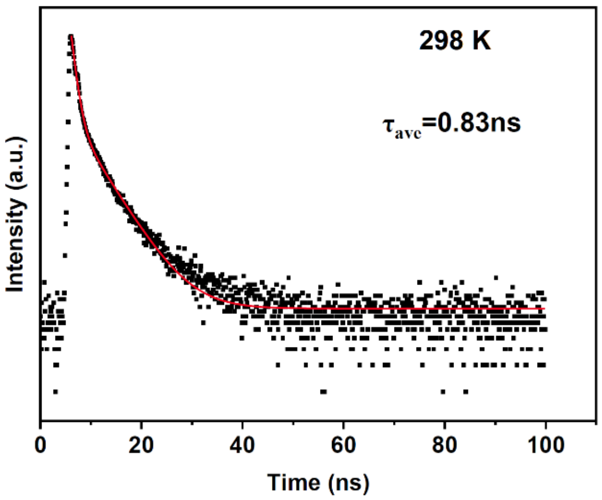

Figure S4. Transient photoluminescence data of **1** at 298 K (excited at 370 nm).

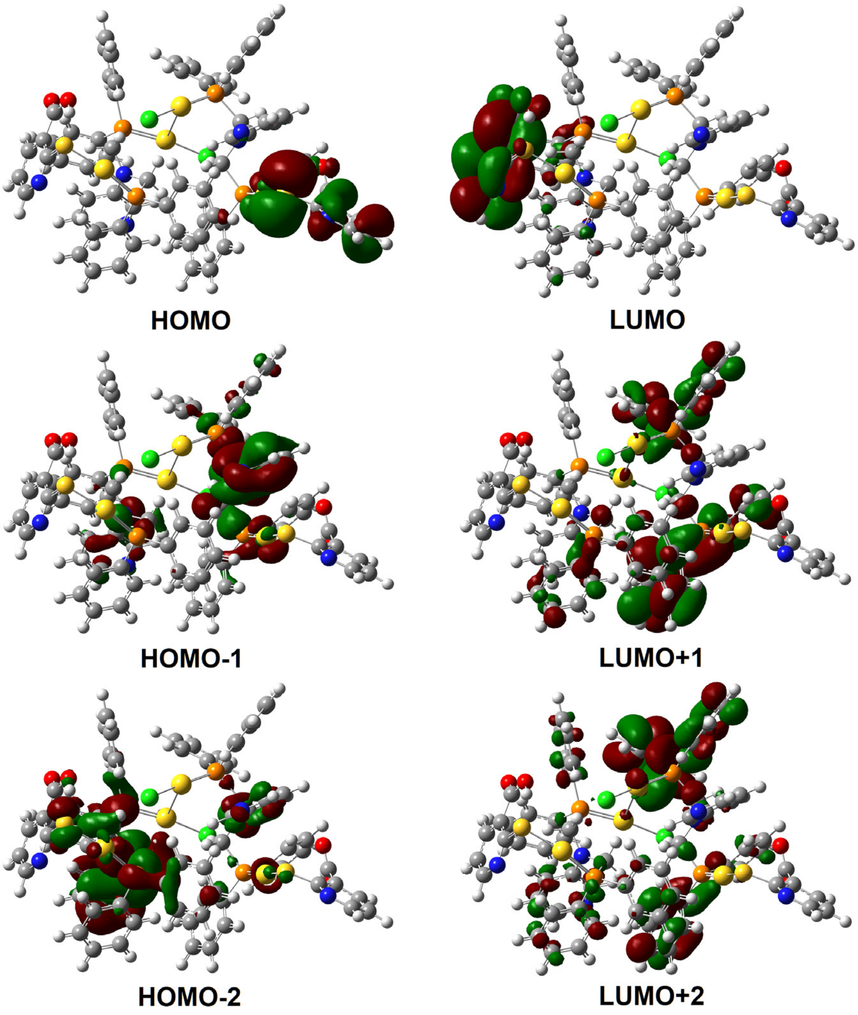

Figure S5. Orbital distributions of HOMOs and LUMOs (isovalue = 0.02) in **1**.

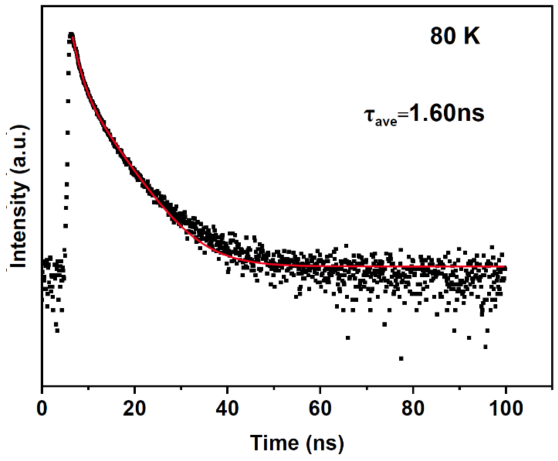

Figure S6. Transient photoluminescence data of **1** at 80 K (excited at 370 nm).

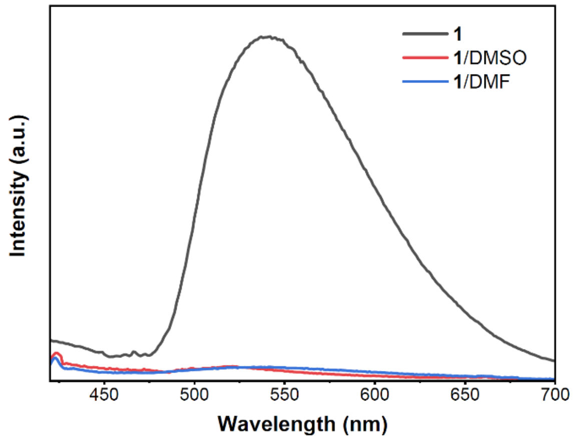

Figure S7. Emission spectra of **1** and the DMSO and DMF solutions of **1** (1 mg/mL, Ex = 377 nm).

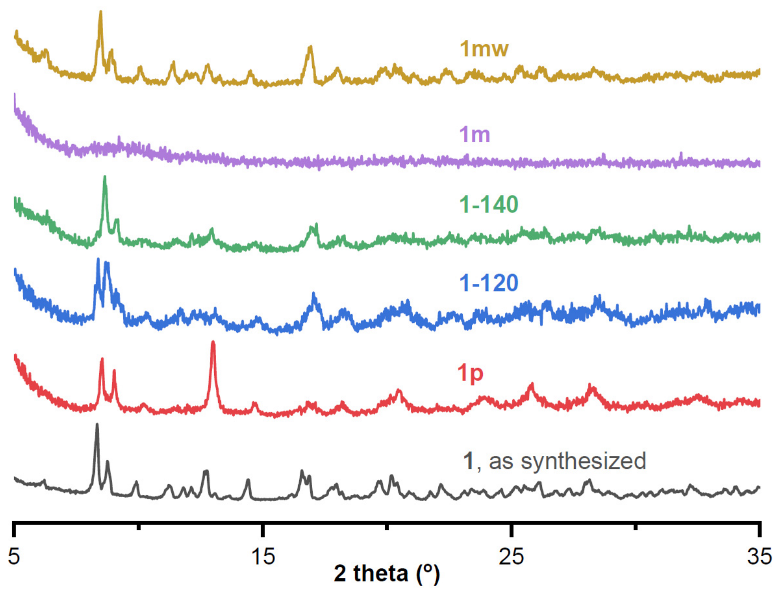

Figure S8. The PXRD patterns of as synthesized **1**, **1p**, **1** heated at 120 °C (**1–120**) and 140 °C (**1–140**), **1m** and **1mw**.

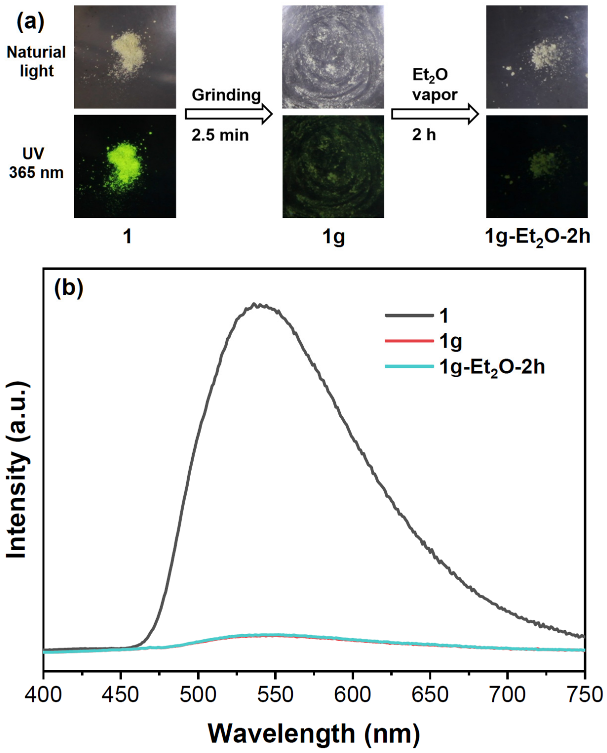

Figure S9. (a) Photographs of **1**, **1g** and **1g** in Et$_2$O vapor for 2h under natural light (upper) and under 365 nm UV light (lower), and (b) their emission spectra (Ex = 377 nm).

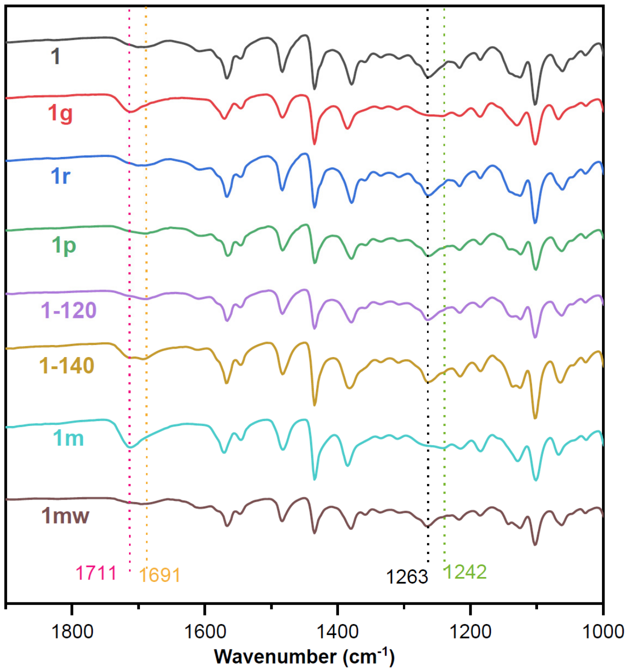

Figure S10. Comparison on the IR spectra in the range of 1000–2000 cm^{-1} of **1**, **1g**, **1r**, **1p**, **1** heated at 120 °C (**1–120**) and 140 °C (**1–140**), **1m** and **1mw**.

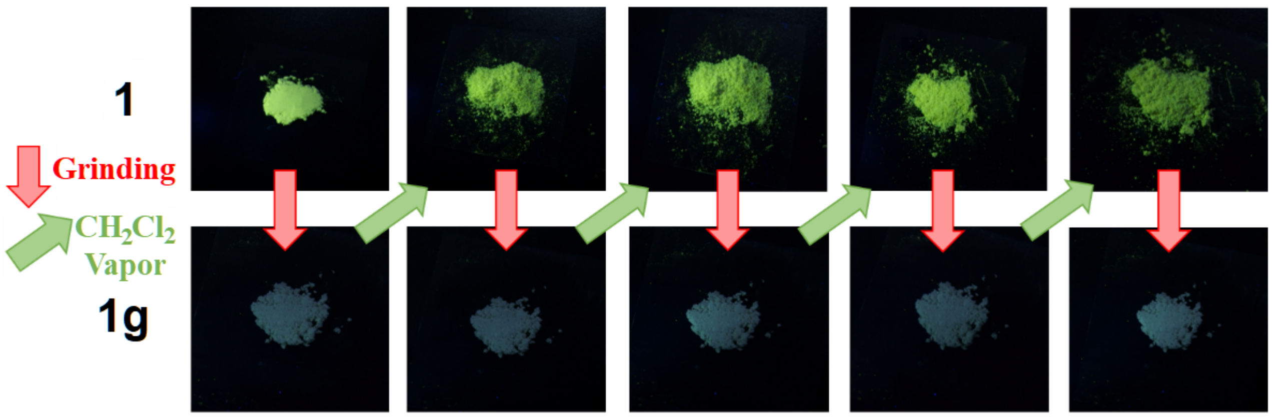

Figure S11. Photographs of **1** (upper) and **1g** (lower) under irradiation at 365 nm over five interconversion cycles. The red and green arrows indicate grinding and exposure to CH_2Cl_2 vapor, respectively.

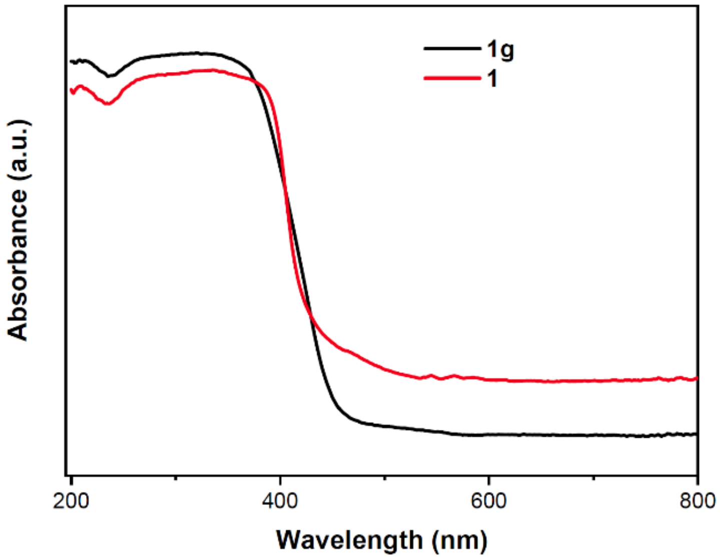

Figure S12. Solid-state UV-Vis spectra of **1** and **1g**.

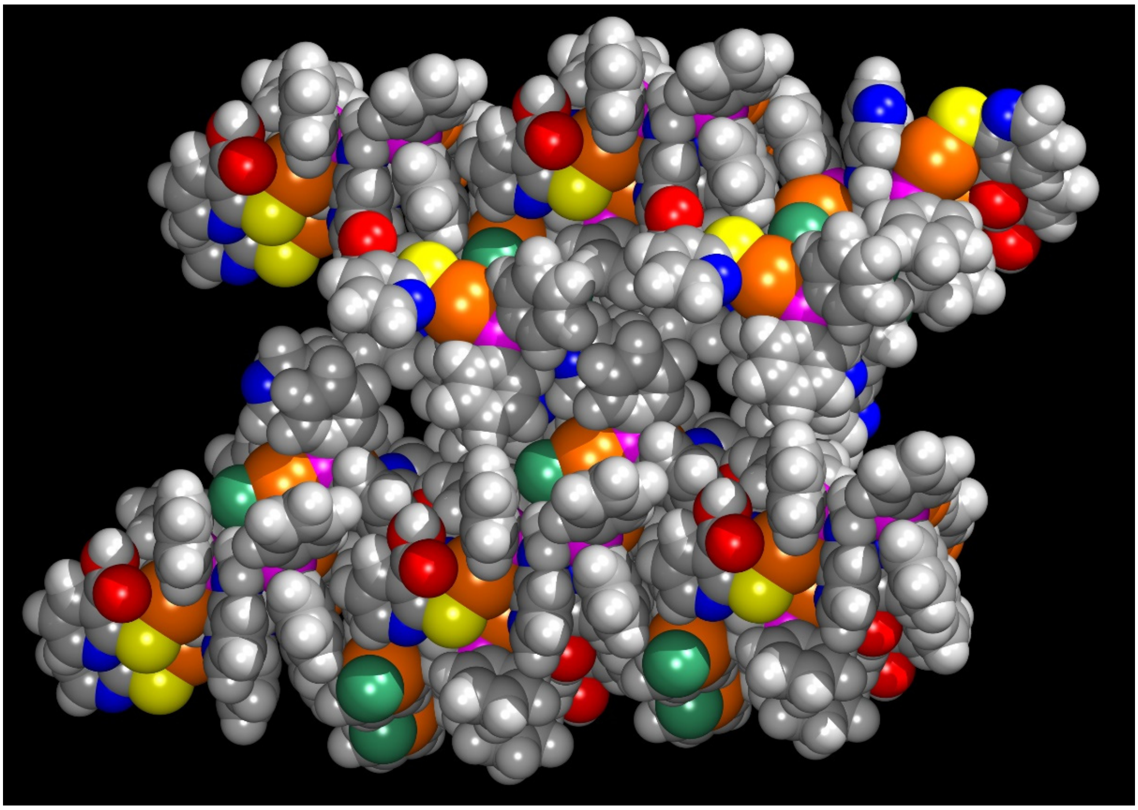

Figure S13. Plot representing the voids in the single crystal of **1**·0.5Et_2O·0.5CH_2Cl_2 after removing all solvates.

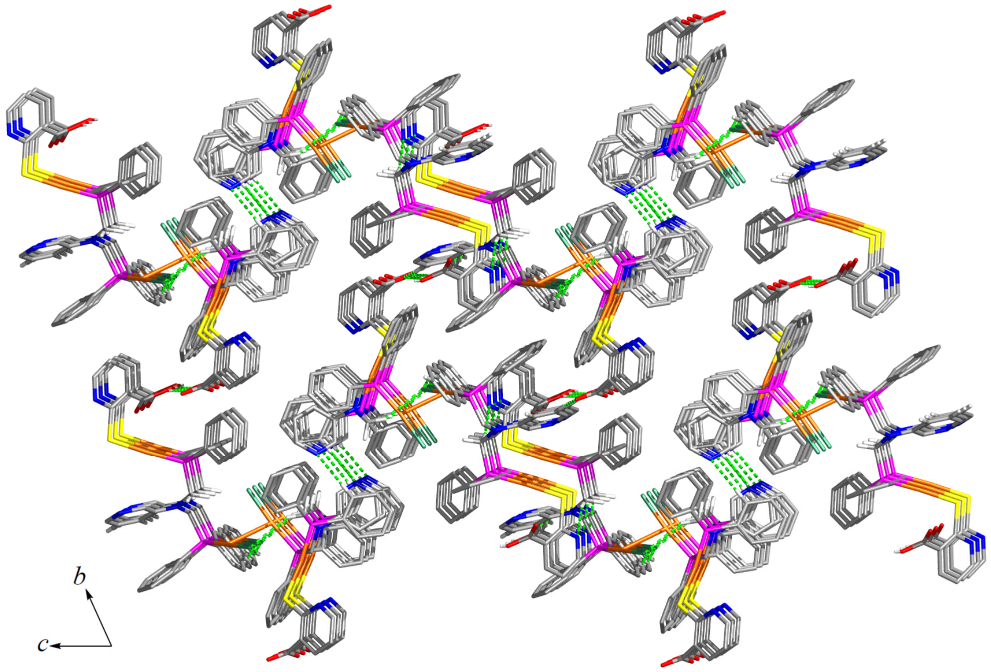

Figure S14. Packing of the main structure of **1** viewed along the *a* axis in the crystal structure of 1·0.5Et_2O·0.5CH_2Cl_2. Hydrogen bonds were plotted as green dash lines. Atom colours: Au, orange; S, yellow; P, purple; Cl, green; N, blue; O, red; C, dark gray; H, light gray.

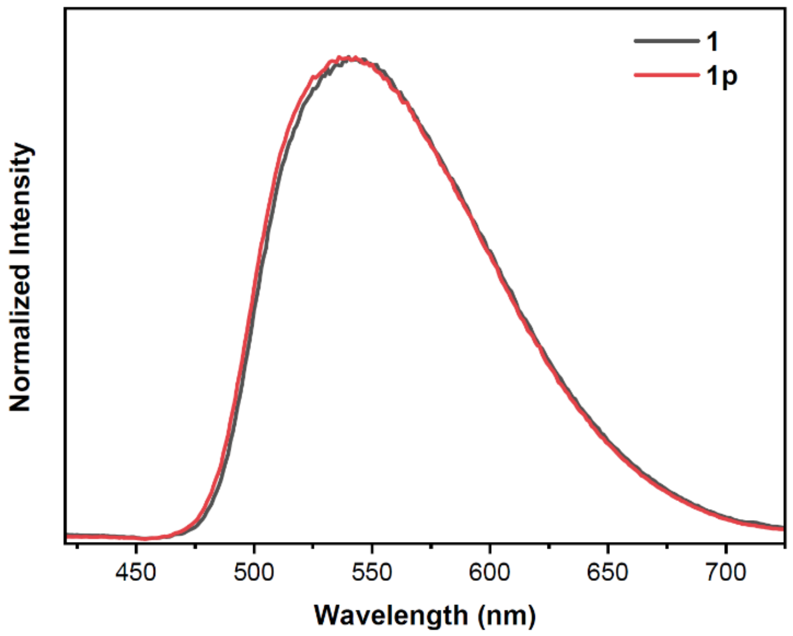

Figure S15. Emission spectra of **1** and **1p** (Ex = 377 nm).

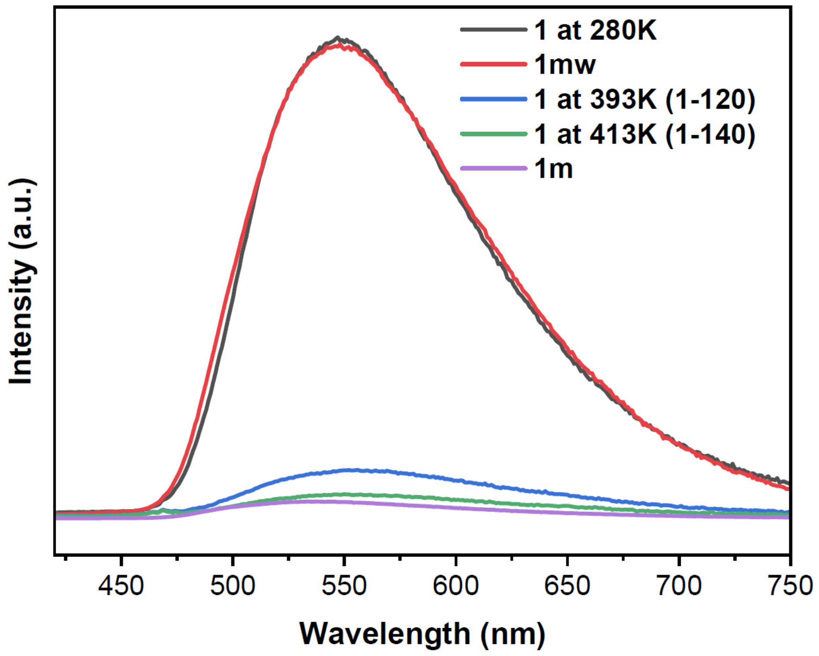

Figure S16. Emission spectra of **1m**, **1mw**, **1** at 280 K, at 393 K (120 °C, **1–120**) and at 413 K (140 °C, **1–140**) (Ex = 377 nm).

Table S1. Emission wavelength (λ_{max}), life time (τ) and quantum yield (QY) of some Au–P complexes in literature.

Formula	λ_{max} (nm)	τ	QY (%)	Ref.
$Au(PPh_3)_2Cl$	560	19 μs	N/A	6
$Au(PPh_3)_2Br$	540	12 μs	N/A	
$(PhCl)_3PAuI$ monoer	472	3.7 ms	4.9	7
$(PhCl)_3PAuI$ dimer	543	0.36 ms	70.5	
$[Au_3(PPh_2CH_2PPhCH_2PPh_2)_2](PF_6)_3$	460	2.4 μs	64	8
$[Au_{10}\{\mu_3\text{-}Ph_2PN(CH_2\text{-}o\text{-}C_5H_4N)PPh_2\}_4(\mu_3\text{-}S)_4](PF_6)_2$	495	μs regime	26	13
$Au_4Cu_2(decz)_2(POP)_2$	565	14.5 μs	50.2	18
$[Au_2(nixantphos)_2](CF_3COO)_2$	596	0.3 μs	N/A	27
Au(XantPhos)Cl	520	19.7 μs	81	34
$[Au(dppb)_2]Cl$	478	3.6 μs	86	37
$[(AuCl)(3\text{-}bdppmapy)(AuCl)_3(3\text{-}bdppmapy)]$	530	4.9 ns	1.8	38
$\{[(3\text{-}bdppmapy)(AuCl)_2]\}_n$	525	4.1 ns	5	
$[(3\text{-}bdppmapy)_2(AuHmba)_3(AuCl)]$	522	2.58 ns	4.7	39
$[Au_2Cu_2(3\text{-}dppmapz)_2](PF_6)_2\cdot 4MeOH$	560	94.8 μs	80	42
$[Au_4(dppmt)_4(AgCl)_2]_n$	495	0.78 μs	22.3	43

Table S2. Selected crystallographic data and refinement parameters for **1**$\cdot 0.5Et_2O\cdot 0.5CH_2Cl_2$.

Formula	$C_{76.5}H_{70}Au_4Cl_3N_6O_{4.5}P_4S_2$
Fw	2227.60
Crystal system	triclinic
Space group	$P\bar{1}$
a/ Å	11.0934(7)
b/ Å	16.5890(10)
c/ Å	22.8267(16)
α/ deg	69.523(3)
β/ deg	78.771(3)
γ/ deg	74.460(3)
V/ Å^3	3767.6(4)
Z	2
ρ_{calc} (g/cm^3)	1.964
μ (mm^{-1})	11.646
F(000)	2132
R_1[a]	0.0594
wR[b]	0.1417
GOF[c]	1.077

[a]$R_1 = \Sigma||F_o| - |F_c||/\Sigma|F_o|$, [b]$wR_2 = \{\Sigma w(F_o^2 - F_c^2)^2/\Sigma w(F_o^2)^2\}^{1/2}$. [c]$GOF = \{\Sigma w((F_o^2 - F_c^2)^2)/(n - p)\}^{1/2}$, where n = number of reflection and p = total number of parameters refined.

Table S3. Selected bond lengths (Å) and angels (°) in **1**·0.5Et_2O·0.5CH_2Cl_2.

Au2–Au3	3.1312(6)
Au1–P1	2.254(2)
Au1–S1	2.305(3)
Au2–P2	2.225(2)
Au2–Cl1	2.286(2)
Au3–P3	2.234(2)
Au3–Cl2	2.300(2)
Au4–P3	2.252(2)
Au4–S2	2.299(2)
P1–Au1–S1	169.89(11)
P2–Au2–Cl1	173.47(9)
P2–Au2–Au3	102.64(6)
Cl1–Au2–Au3	82.25(6)
P3–Au3–Cl2	177.37(8)
P3–Au3–Au2	97.56(6)
Cl2–Au3–Au2	84.66(5)
P4–Au4–S2	175.41(9)
